# Time-of-flight PET/CT suppresses CT based attenuation correction and scatter coincidence correction errors due to misalignment of the gastrointestinal tract

**DOI:** 10.22038/AOJNMB.2024.74406.1520

**Published:** 2024

**Authors:** Yuya Watanabe, Shota Hosokawa, Yasuyuki Takahashi

**Affiliations:** Hirosaki University Graduate School of Health Sciences, Hirosaki-shi, Aomori, Japan

**Keywords:** Time-of-flight technique, Positron emission, tomography, Computed tomography-based attenuation correction

## Abstract

**Objective(s):**

This study aimed to examine the influence of changes in CT values on PET images, specifically focusing on errors in CT-based attenuation correction and scatter coincidence correction (CTAC/SC) caused by gastrointestinal gas. Furthermore, it aimed to demonstrate the effectiveness of time-of-flight (TOF) PET in reducing CTAC/SC errors.

**Methods:**

PET images were reconstructed using multiple CT images with varying CT values. The study then compared the fluctuations in pixel values of the PET images corresponding to the different CT values utilized for CTAC/SC between non-TOF and TOF acquisitions.

**Results:**

PET pixel values fluctuated with changes in CT values. In the phantom study, TOF showed a significantly smaller change in PET pixel value of 1.00±0.27 kBq/mL compared to 3.72±1.33 kBq/mL in the non-TOF at sites with a CT change of +1000 HU. In the patient study, a linear regression analysis was performed to determine the effect of changes in CT values due to gastrointestinal gas migration on standard uptake value (SUV).The results showed that the TOF group had a lower ratio of change in SUV to change in CT values compared to the non-TOF group. These findings revealed that PET pixel values exhibited fluctuations in response to changes in CT values, and TOF-PET effectively mitigated CTAC/SC errors arising from gastrointestinal gas.

**Conclusions:**

TOF-PET has the potential to reduce the occurrence of suspicious accumulation.

## Introduction

 Positron emission tomography (PET) is mainly used to examine malignant tumors using ^18^F-fluoro-2-deoxy-d-glucose (^18^F-FDG). 

 PET/computed tomography (CT) improves diagnostic capabilities by combining functional and morphological information from PET and CT imaging of the same area, respectively, in a single examination (1).　Most PET/CT systems use CT-based attenuation correction and scatter coincidence correction (CTAC/SC), which corrects γ-ray attenuation and scatter coincidence correction using CT images (2, 3). 

 Therefore, misregistration between CT and PET may affect the diagnosis through visual image misalignment and degradation of image quality and quantification. Since the examinations are performed sequentially, a difference is observed between the start times of PET and CT. Previously, poor quantification and CTAC/SC errors in PET images due to misregistration between the two images caused by respiratory (4, 5), cardiac (6, 7), body, and peristaltic motions have been reported. 

 Gastrointestinal tract motions are difficult to address as they are involuntary and non-cyclical. Nakamoto et al. reported that peristalsis causes over- or underestimation of the standard uptake value (SUV) in comparison with CTAC/SC and attenuation correction using a ^68^Ga source (8). Lodge et al. reported that high-intensity artifacts due to gas movement occur in the posterior bladder around the rectum (9). 

 Differentiation of artifacts related to the gastrointestinal tract can be achieved by delayed scanning (10); however, this confers disadvantages to patients, such as increased exposure and time constraints. In time-of-flight (TOF) PET, the approximate onset point of radioisotopes (RIs) can be determined by calculating the difference in the time of arrival of annihilation γ-rays to opposing detectors (11). This reduces noise and improves the signal-to-noise ratio (SNR) of PET images (12, 13). Furthermore, TOF-PET provides a more accurate distribution of RIs compared with conventional PET. Conti reported a reduction in attenuation correction artifacts with the use of TOF (14), while Son et al. reported improve-ments in various inaccurate data corrections (15). Based on these reports, we hypothesized that TOF may suppress CTAC/SC errors caused by the misalignment of the gastrointestinal tract between PET and CT.

 Therefore, this study aimed to demonstrate that TOF has a suppressive effect on CTAC/SC errors caused by the misalignment of the gastrointestinal tract between PET and CT. 

 Simulating the complex and time-varying motion of the intestines is incredibly challenging. In our previous research, we demonstrated that quantitative values in PET can be influenced by differences in CT imaging materials, particularly in cases where there are variations in CT values of the constituent materials, leading to significant CTAC/SC errors in areas containing air (16). Consequently, in this study, we focused on the gastrointestinal gas and compared the effects of altering the location of gastrointestinal gas in CT images on PET images between TOF and non-TOF acquisitions.

## Methods

 The PET/CT system used in this study was Discovery MI (GE Healthcare, Milwaukee, Wisconsin), the latest generation PET/CT scanner with silicon photomultiplier (SiPM) detectors. The CT scanner was a 64-row multislice scanner, and the imaging conditions were as follows: tube voltage, 120 kVp; X-ray tube current, 140 mA (phantom study) and auto mA (patient study); slice thickness, 3.75 mm; revolution time, 0.984 s; and spiral pitch factor, 0.35 s. The PET detector comprised a lutetium-based scintillator (LBS), four detector rings, a crystal size of 3.95×5.3×25 mm, 19,584 crystals, a detector ring diameter of 740 mm, a transaxial field of view of 700 mm; and axial field of view 200 mm. The coincidence time resolution (CTR) was 385 ps. PET images were reconstructed using the three-dimensional ordered subset expectation maximization (3D-OSEM) method (VUE Point HD) and OSEM+TOF (VUE Point FX). 

 The PET reconstruction conditions were as follows: iterations, 4; subsets, 16; and matrix size, 256×256, using a standard Z-axis filter and full width at half maximum (FWHM) of the Gaussian filter of 5.0 mm. These conditions were used for both the phantom study and the patient study.

***Phantom study***

 In the phantom study, we investigated the impact of substituting a different substance during CT imaging at the locations within the phantom that were initially air-filled during PET imaging. Specifically, we examined how the variations in CT values resulting from this substitution affected the PET image. The abdominal region exhibits a wide range of CT values due to the presence of various tissues such as soft tissue, visceral fat, as well as gas and contents within the gastrointestinal tract. 

 Therefore, several changes in the CT values in the range from air (-1000 HU) to water (0 HU) were considered for verification. A National Electrical Manufacturers Association (NEMA) International Electrotechnical Commission (IEC) body phantom was used in the phantom study. Six spheres with diameters of 10, 13, 17, 22, 28, and 37 mm were placed in a noncircular outer container, and a cylinder resembling a lung was inserted into the center. The phantom background was filled with 2.65 kBq/mL of ^18^F-FDG solution, and each inserted sphere was filled with non-radioactive air, assuming the presence of gas in the gastrointestinal tract. PET acquisition was performed for two minutes following CT imaging. CT images with different CT values were generated based on the same raw PET data and used in CTAC/SC to generate PET images with altered CT values, following the procedure described as follows. ImageJ version 1.52, an open-source image analysis software (17), was used to change the CT values of the sphere areas. The pixel values of the CT images were modified in increments of 200 HU, ranging from -1000 HU to 0 HU, resulting in the creation of six series of CT images. These series, collectively referred to as the "edited CT." Each of the different series within the edited CT, characterized by their respective pixel values, was used for CTAC/SC to reconstruct PET images. Two sets of reconstructed PET images were generated, one with TOF and the other without TOF. These reconstructed PET images were then referred to as the "edited PET." The CT where the CT value of the sphere is set to air (-1000 HU) is referred to as the "reference CT. 

**Figure 1:**
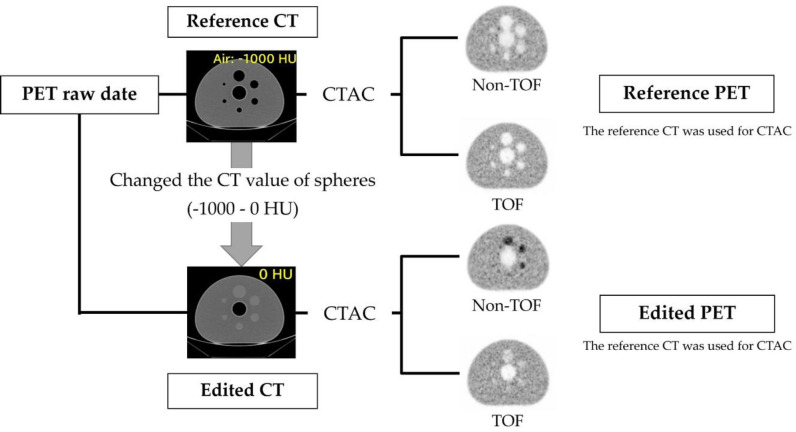
The image reconstruction procedure in phantom studies. Positron emission tomography (PET) images were reconstructed using computed tomography (CT)-based attenuation correction (CTAC/SC) for each of the six series of CTs with different CT values in the sphere on the same PET raw data. The 'edited CT' refers to the CT image where the CT value of the sphere was modified. The 'edited PET' refers to the PET image reconstructed using the edited CT. The 'reference CT' refers to the CT image where the CT value of the sphere was adjusted to -1000 HU (air). The 'reference PET' refers to the PET image reconstructed using the reference CT”

 Furthermore, the PET images reconstructed using the reference CT were considered as the "reference PET." Figure 1 illustrates the image reconstruction procedure in phantom studies. 

 At each CT value change, the regions of interest (ROIs) with the same size as the spheres were drawn on the CT image and copied to the PET image, and the average pixel value (Bq/mL) was calculated. The change in CT values, ΔHU_phantom_, within the ROI was determined using the following equation:

${\mathrm{\Delta HU}}_{\mathrm{phantom}}={\mathrm{HU}}_{\mathrm{edited}}-{\mathrm{HU}}_{\mathrm{reference}}$ (1)

 Where, HU_edited_ represents the pixel value of the edited CT within the ROI, and HU_reference _represents the pixel value of the reference CT that was changed the CT value of the sphere to -1000 HU within the ROI. The change in PET values, ΔPET, within the ROI was determined using the following equation:

$\mathrm{\Delta PET}={\mathrm{PET}}_{\mathrm{edited}}-{\mathrm{PET}}_{\mathrm{reference}}$ (2)

 Where, PET_edited_ represents the pixel value of the edited PET within the ROI, and PET_reference_ represents the pixel value of the reference PET within the ROI. The aforementioned procedure was executed for both non-TOF and TOF reconstructions, and the relationship between ΔHU_phantom_ and ΔPET for each reconstruction method was evaluated.

 In order to simulate tumors, the NEMA IEC Body phantom was subjected to comparable conditions. The phantom background was filled with 2.65 kBq/mL of ^18^F-FDG solution, and each inserted sphere was filled with an ^18^F-FDG solution at four times the background radiation concentration. Subsequently, PET acquisition was performed for two minutes. An edited CT was generated by altering the pixel values within the inserted sphere of the CT images in increments of 200 HU from -1000 HU to 0 HU. 

 These edited CTs were utilized for correction to produce edited PET images. ROI of the same size as the sphere was delineated on the CT images, and this ROI was copied onto the PET images to calculate the mean pixel value (Bq/mL). Using equations (1) and (2), the ΔHU_phantom_ and ΔPET for the simulated tumor region were determined. A subsequent comparison was performed between non-TOF and TOF.

***Patient study***

 In total, 16 participants (eight males and eight females) who underwent PET/CT examinations between October 2021 and August 2022 were included in the analysis. In this study, all 16 patients underwent CT re-imaging after PET imaging in addition to routine CT imaging. 

 Typically, CT imaging is performed before PET imaging, but in cases where a mismatch occurs due to differences in respiratory phase between PET and CT imaging, a repeat CT scan may be conducted. The patient selection process involved comprehensive criteria, including the absence of a history of hyperglycemia, diabetes mellitus, or liver disease, as well as the absence of significant accumulations in the abdominal region. The characteristics of the participants were as follows: mean age, 71.8±10 years (42–85 years); weight, 70.5±9.9 kg (42.0–84.0 kg); and body mass index (BMI), 23.7±3.0 kg/m^2^ (18.7–31.3 kg/m^2^). In addition, the dose was 274.3±55.0 MBq (165.5–344.3 MBq). The duration of fasting was at least 4 hours, and the waiting period between ^18^F-FDG administration and the start of PET acquisition was 60 minutes to allow for urinary drainage and reduce accumulation in the urinary tract system immediately before imaging. The acquisition time ranged from 1 minute and 30 seconds to 3 minutes per bed, with the acquisition times varying depending on the BMI and dose. This study was conducted after obtaining certification from the Ethics Committee of Hirosaki University School of Medicine (Certification number: 202117), and was performed in accordance with the ethical standards laid out in the 1964 Declaration of Helsinki.

**Figure 2:**
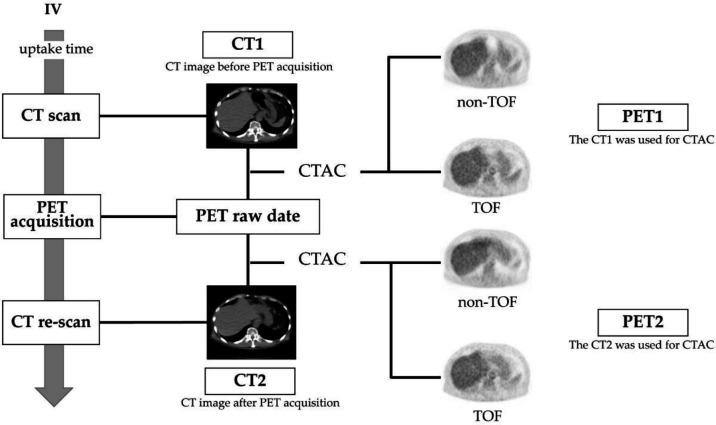
Image reconstruction procedure in patient study. Positron emission tomography (PET) images were reconstructed using computed tomography (CT)-based attenuation correction (CTAC/SC) with different computed tomography (CT) images from the same raw PET data. The 'CT1' refers to the CT image before PET acquisition. The 'PET1' refers to the PET image reconstructed using the CT1. The 'CT2' refers to the CT image after PET acquisition. The 'PET2' refers to the PET image reconstructed using the CT2

 In this study, the CT images acquired before PET imaging were referred to as CT1, and the CT images acquired after PET imaging were referred to as CT2. Using the same raw PET data, the PET image reconstructed using CT1 was referred to as PET1, and the PET image reconstructed using CT2 was referred to as PET2. Figure 2 illustrates the image reconstruction procedure employed in patient studies. We examined the effect of tissue change at the gastrointestinal gas present location on the standardized uptake value (SUV) of PET images. The investigation focused on the impact of the differences in CT values on the SUV of the PET images. To define the ROI in the location of gastrointestinal gas in CT1, a thresholding technique was employed. The threshold was set within the CT value range of -1100 to -900 HU to match the gas in the gastrointestinal tract. 

 The Wand (Trace) tool, functionality in ImageJ, was utilized to outline the ROI along the contours. The ROI was obtained for each slice from the superior border of the liver to the pubic symphysis. The effective size of the ROI was more than 200 mm^2^. The ROI from CT1 was copied to CT2, and the average pixel values within the ROIs of CT1 and CT2 were measured. 

**Figure 3:**
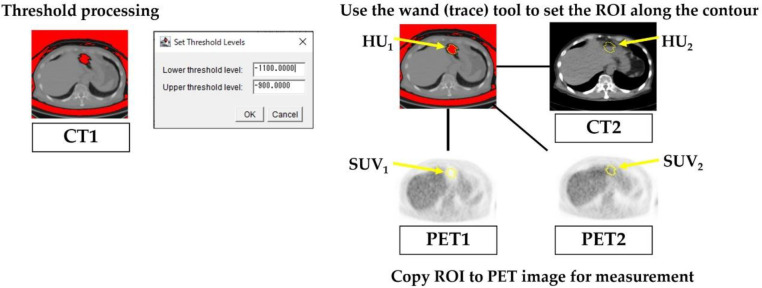
Procedure for setting the regions of interest (ROI). Using thresholding, an ROI was placed at the location of the gastrointestinal gas in CT1, and this ROI was then copied onto the other images

Figure 3 illustrates the procedure for setting the ROI. Then, the change in CT values, ΔHU_patient_, was calculated using the following equation:

${\Delta \mathrm{HU}}_{\mathrm{patient}}={\mathrm{HU}}_{2}-{\mathrm{HU}}_{1}$ (3)

 Where, HU_1_ represents the pixel values within the ROI in CT1, and HU_2_ represents the pixel values within the ROI in CT2. The ROI from CT1 was copied to PET1 and PET2, and the mean pixel values within the ROIs of PET1 and PET2 were measured. The mean pixel value was converted to mean SUV (g/mL) using the following formula:

$\mathrm{SUV}=\frac{\mathrm{activity concentration}}{\frac{\mathrm{injected dose}}{\mathrm{body weight}}}(g/\mathrm{mL})$ (4)

 The change in SUV within the ROI, ΔSUV, was calculated using the following equation:

$\mathrm{\Delta SUV}={\mathrm{SUV}}_{2}-{\mathrm{SUV}}_{1}$ (5)

 Where, SUV_1_ represents the average SUV within the ROI in PET1, and SUV_2_ represents the average SUV within the ROI in PET2. The aforementioned procedure was executed for both non-TOF and TOF reconstructions, and the relationship between ΔHU_patient_ and ΔSUV for each reconstruction method was evaluated.

***Statistical Analysis***

 In the phantom study, data are expressed as mean ± standard deviation. Non-TOF and TOF ΔPET were compared using paired t-tests. In the patient study, linear regression analysis using least squares and Spearman's rank correlation coefficient ρ were employed to determine the association between ΔHU_patient_ and ΔSUV. 

 Statistical significance was set at p-value < 0.05. All statistical analyses were performed using python (version 3.11) with the utilization of the pandas, scipy, and matplotlib libraries.

## Results

**Figure 4:**
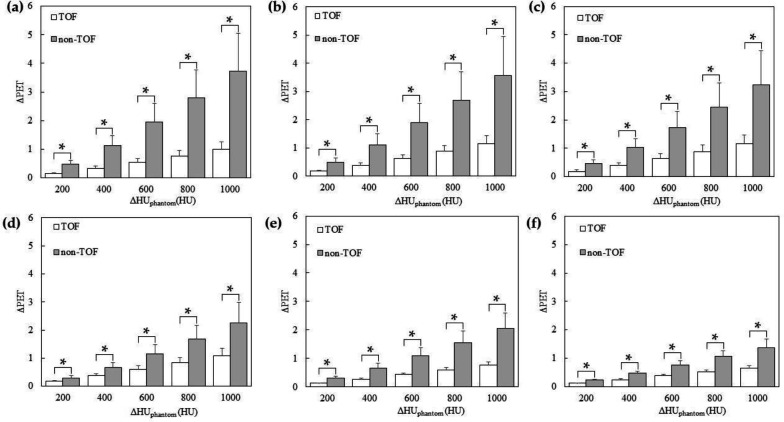
Relationship between ΔHU_phantom_ and ΔPET for the validation with different computed tomography (CT) values in the sphere area. The sizes of the insert spheres are 37 mm (**a**), 28 mm (**b**), 22 mm (**c**), 17 mm (**d**), 13 mm (**e**), and 10 mm (**f**). Statistically significant differences with p < 0.05 are marked with single asterisk

**Figure 5:**
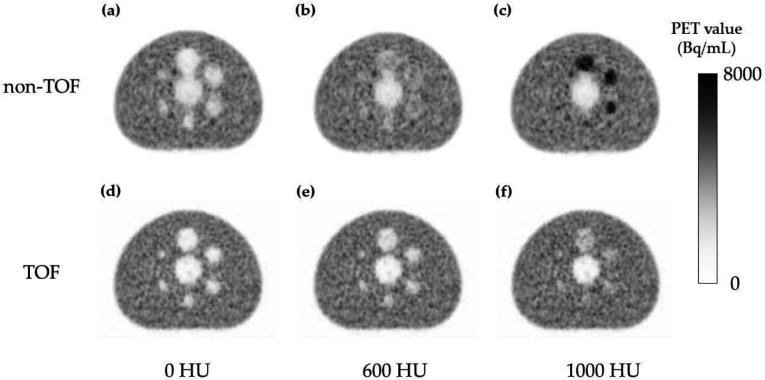
Positron emission tomography (PET) images for validation with different computed tomography (CT) values in the sphere area. Non-TOF: ΔHU_phantom_ of 0 HU (**a**), ΔHU_phantom_ of 600 HU (**b**), and ΔHU_phantom_ of 1000 HU (**c**). TOF: ΔHU_phantom_ of 0 HU (**d**), ΔHU_phantom_ of 600 HU (**e**), and ΔHU_phantom_ of 1000 HU (**f**)

Figure 4 shows the relationship between ΔHU_phantom _and ΔPET for the validation with different CT values of the spheres in the phantom study. ΔPET of all spheres tended to increase with increasing ΔHU_phantom_. ΔPET relative to ΔHU_phantom_ was significantly smaller in the TOF compared with that in the non-TOF. Figure 5 shows the PET images used for validation with the changed CT values of the spheres. Non-TOF showed that the sphere was indistinguishable from the background at ΔHU_phantom_ of 600 HU (Figure 5b), whereas the sphere was clearly visible as a positive signal at ΔHU_phantom_ of 1000 HU (Figure 5c). 

 In contrast, in TOF, the sphere was identified as a negative signal at both ΔHU_phantom_ of 600 HU (Figure 5e) and ΔHU_phantom_ of 1000 HU (Figure 5f).

**Figure 6:**
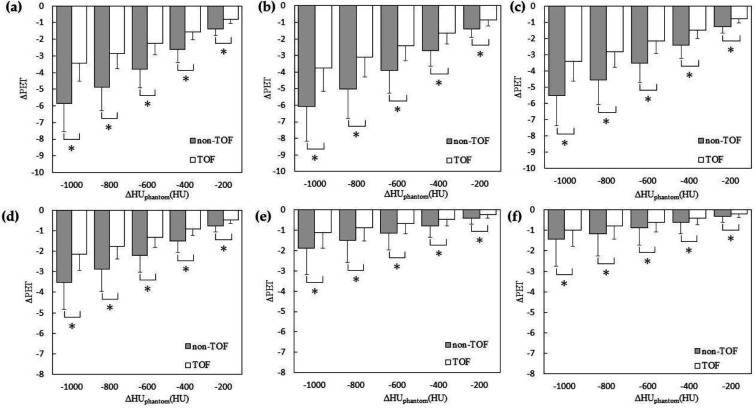
Relationship between ΔHU_phantom_ and ΔPET in tumor-simulating phantom validation for the validation with different computed tomography (CT) values in the sphere area. The sizes of the insert spheres are 37 mm (**a**), 28 mm (**b**), 22 mm (**c**), 17 mm (**d**), 13 mm (**e**), and 10 mm (**f**). Statistically significant differences with p < 0.05 are marked with single asterisk

**Figure 7:**
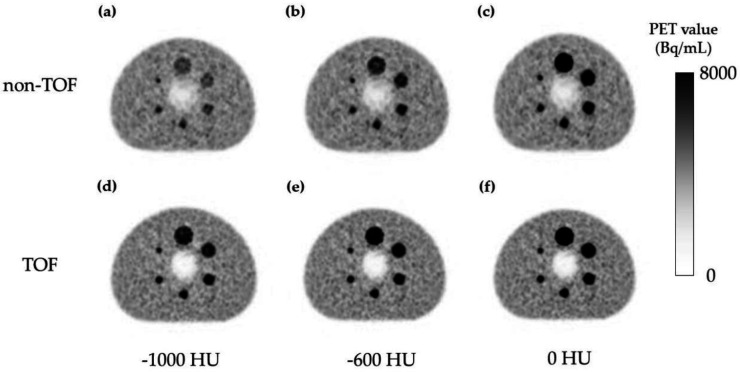
Positron emission tomography (PET) images for validation with different computed tomography (CT) values in the sphere area in simulated tumor phantom verification. Non-TOF: ΔHU_phantom_ of -1000 HU (**a**), ΔHU_phantom_ of -600 HU (**b**), and ΔHU_phantom_ of 0 HU (**c**). TOF: ΔHU_phantom_ of -1000 HU (**d**), ΔHU_phantom_ of -600 HU(**e**), and ΔHU_phantom_ of 0 HU (**f**)

Figure 6 shows the relationship between ΔHU_phantom_ and ΔPET when validating the simulation of tumors using spheres with varied CT values in the phantom study. There was an observed tendency for ΔPET to decrease with decreasing ΔHU_phantom_ across all accumulations in the spheres. Notably, the ΔPET relative to ΔHU_phantom_ was significantly smaller in TOF compared to non-TOF. Figure 7 shows the PET images used for the validation of the tumor-simulated phantom with altered CT values for the spheres are presented. In the non-TOF images, at ΔHU_phantom_ of -1000 HU (Figure 7a), a decrease in accumulation was evident for all spheres, with a marked reduction in accumulation for the 28 mm and 37 mm spheres. In contrast, the TOF images maintained clear accumulations even at ΔHU_phantom_ of -1000 HU (Figure 7d).

**Figure8:**
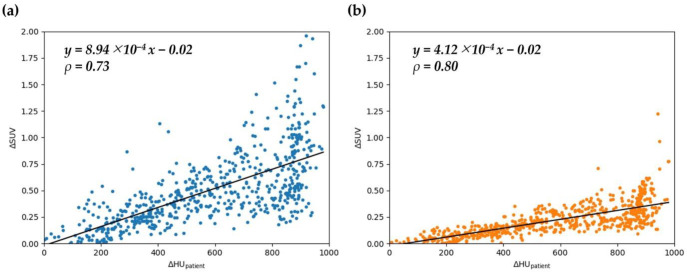
Relationship between ΔHU_patient_ and ΔSUV in the patient study. Non-TOF group (**a**), and TOF group (**b**). *ρ* is the Spearman's rank correlation coefficient

Figure 8 shows the relationship between ΔHU_patient_ and ΔSUV in the patient study. In the non-TOF group, a regression line of y=8.98×10^-4 ^x-0.02 was obtained, and the Spearman's correlation coefficient ρ was 0.73 (p < 0.05). In the TOF group, a regression line of y=4.12×10^-4 ^x-0.02 was obtained, and the Spearman's correlation coefficient *ρ* was 0.80 (p < 0.05). 

**Figure 9:**
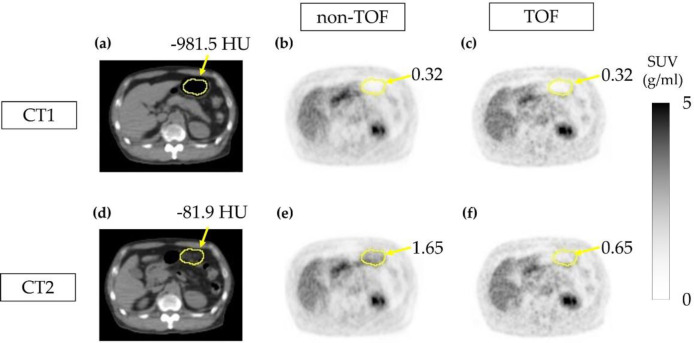
Positron emission tomography (PET) images in the patient study. CT1 (**a**), PET1 in the non-TOF group (**b**), and PET1 in the TOF group (**c**). CT2 (**d**), PET2 in the non-TOF group (**e**), and PET2 in the TOF group (**f**)

Figure 9 shows an example of a PET image. In the region where misalignment occurred, HU_1_ was -981.5 HU and HU_2_ was -81.9 HU, resulting in a ΔHU_patient_ of 899.6 (Figure 9a,d). In the non-TOF group, SUV was 0.32 g/mL in PET1 and 1.65 g/mL in PET2, indicating an increase in signal intensity (Figure 9b,c). The visual accumulation not seen in PET1 was observed in PET2. In the TOF group, SUV was 0.32 g/mL in PET1 and 0.65 g/mL in PET2, indicating an increase in signal intensity (Figure 9e,f). The visual accumulation was not seen in either PET1 or PET2.

## Discussion

 Phantom and patient studies were conducted in the present study to demonstrate the suppression of the artifacts caused by CTAC/SC errors in the gastrointestinal tract by TOF-PET. 

 In the phantom study, assuming the replacement of the contents of the gastro-intestinal tract, CT images with different CT values were created and used for CTAC/SC to reconstruct the PET images. The changes in the PET pixel values due to different CT values were investigated. As a result, ΔPET increased according to the changes in the CT values. In CTAC/SC, CT values are converted to line attenuation coefficients at 511 keV γ-rays (μ511 keV). The PET pixel values changed in accordance with changes in CT values due to the differences between the μ511keV of the constituent material during actual PET imaging and the μ511keV calculated from the CT by CTAC/SC, resulting in overcorrection compared with the original attenuation correction according to this difference (16). The results of this study were consistent with our previous research. In the previous study, a phantom was utilized, featuring a cylindrical structure with a diameter of 20mm and enclosing a test tube of 15 mm in diameter. It was recognized that assuming a torso region was challenging due to the cylindrical shape and small diameter of the phantom. On the other hand, the NEMA IEC body phantom used in this study is designed to replicate the abdominal section, expressing the torso region with a shape that simulates the human abdominal area. This allows for a more clinically relevant condition. Additionally, while the acquisition time was set to 30 minutes in the previous study, this research adopted the commonly used clinical condition of 2 minutes, aligning with conditions widely employed in clinical settings. Compared with that in the non-TOF group, ΔPET was significantly reduced in the TOF group. This was because, as Conti suggested, the spatial weighting derived from the TOF information during reconstruction partially corrected the accumulation of activity in parts of the image that were inconsistent with the data timing information (14). The TOF-PET system provided information on the spatial location of the RI. TOF indicated that the amount of RI in the sphere was low in the spheres filled with air with no RIs. TOF acted as if it suppressed CTAC/SC errors, even when the LOR counts increased with CT values due to CTAC/SC errors. Thus, although TOF was not directly involved in CTAC/SC, it reduced the CTAC/SC error by reflecting the distribution of the RI at the time of PET acquisition. ΔPET tended to increase as the size of the sphere increased; however, the difference was statistically significant for all spheres, suggesting that TOF was effective even in smaller spheres of 10 mm since it reduced the CTAC/SC error compared with non-TOF. 

 Currently, TOF is standardly utilized, with the majority being based on photomultiplier tubes (PMT) detectors. The SiPM detectors used in this study possesses high TOF performance, which may have resulted in a more substantial artifact suppression effect.

 In the phantom study simulating tumors, the accumulation in PET images decreased with a reduction in CT values in the CT images of the accumulative region. However, the TOF group exhibited a lesser proportionate decrease in accumulation. The pronounced reduction observed in the 28 mm and 37 mm spheres in the non-TOF group is attributed to the larger size of the region with altered CT values. In the tumor region, TOF demonstrated greater robustness, suppressing the decrease in accumulation even when there was displacement of gas during CT imaging at the position of the tumor observed during PET imaging.

 Patients who underwent CT scans before and after PET acquisition were included in the patient study. The same raw PET data were used to reconstruct two series of PET images using the two series of CTs for CTAC/SC. The differences in the CT values of the CT images between the two series and the differences in SUV of the PET images were investigated subsequently. As a result, the PET SUV increased with changes in the CT values, as in the phantom study. Comparing the TOF group to the non-TOF group, it was observed that the TOF group had a smaller slope for the regression line of ΔSUV against ΔHU_patient_. 

 This indicates that the change in SUV in response to CT value variations is smaller in the TOF group. PET1 and PET2 utilized the same raw data, and other corrections were also applied uniformly. Therefore, the difference in SUV between the two PET images is attributed to CTAC/SC. The results of this study suggest that TOF effectively mitigates the impact on SUV by reducing CTAC/SC errors resulting from variances in gastrointestinal gas distribution. In the patient study, a false accumulation due to changes in CT values was observed in the non-TOF group (Figure 9e). Such pseudo-accumulations were observed in other cases, but most of them appeared to be physiologic accumulations of the gastrointestinal tract and could not be mistaken for tumors. Gases are present along the gastrointestinal tract. 

 CTAC/SC errors caused by the migration of gas in the gastrointestinal tract would appear to be physiological accumulation because of the increased accumulation along the gastro-intestinal tract. Tumor-like accumulation would be observed if the gastrointestinal tract gas was spherical, as in the phantom study; if the contents of the displaced gastrointestinal tract were solid, the accumulation would be similar to a tumor. Although there was an increase in the SUV in response to the increased CT values in the TOF group, it did not lead to the presence of accumulation in the image, indicating the possibility of reducing suspicious accumulation.

 Lois et al. reported that light source artifacts appearing over the liver and spleen due to respiratory mismatch between CT and PET are less visible on TOF images (18). Iagara et al. reported that visualization of dental metals, respiratory artifacts, and artifacts due to high FDG excretion to the bladder showed promising results indicating that TOF PET/MRI reduces various PET artifacts (19). The previous studies have not reported any instances of improving the impact of the digestive tract using TOF. 

 Furthermore, these prior studies have focused on noticeable artifacts in PET images, but artifacts resulting from gastrointestinal motion are often difficult to distinguish from physiological accumulations and frequently go unnoticed. The findings obtained in this study, demonstrating the efficacy of TOF in suppresses CTAC/SC errors due to misalignment of the gastrointestinal tract, underscore the utility of TOF beyond visual improvements previously observed in past research, such as artifact reduction, enhanced SNR, and improved spatial resolution. With recent advancements in reconstruction techniques and developments in deep learning, resulting in a clearer delineation of anomalies in PET images, the exclusion of suspicious accumulations becomes increasingly crucial. Especially in areas involving movement within the abdominal region, to fully harness such technologies in clinical and research applications, it is imperative to base them on more precise PET images. High-performance TOF is anticipated to play a particularly crucial role in this regard. While there are studies using deep learning from non-TOF to achieve image quality comparable to TOF (20), the authors emphasize the utility of TOF from a hardware perspective that cannot be solely attained through non-TOF approaches.

 Looking forward, future prospects include leveraging the differences in CTAC errors between non-TOF and TOF to develop methods for detecting displacement in gastrointestinal locations or creating attenuation correction maps with minimal CTAC errors. 

 This study had several limitations. First, CTAC/SC was carried out using the algorithm developed by GE Healthcare (Milwaukee, Wisconsin) and the CT tube voltage was fixed at 120 kV. Second, the effect was expected to vary depending on the temporal resolution of TOF, which was performed using a single device in this study. Lastly, the evaluation of CTAC/SC errors in tumors is clinically important; however, this could not be verified as none of the patients with gastrointestinal tumors underwent two CT scans.

## Conclusion

 In this study, TOF was observed to significantly suppressed CTAC/SC errors due to misalignment of the gastrointestinal tract. This suggests the potential for enhancing the accuracy of PET scans in the abdominal area by suppressing any potential artifacts that might not be visually apparent. The ability of TOF to decrease suspicious accumulations implies the potential to maximize the benefits of future image quality improvement technologies.
